# Dynamic Response and Energy Conversion of Coupled Cantilevers with Dual Piezoelectric–Triboelectric Harvesting Mechanisms

**DOI:** 10.3390/mi16020182

**Published:** 2025-01-31

**Authors:** Mohammad Alghamaz, Leila Donyaparastlivari, Alwathiqbellah Ibrahim

**Affiliations:** 1 Department of Mechanical Engineering, The University of Texas at Tyler, 3900 University Blvd., Tyler, TX 75799, USA; malghamaz@patriots.uttyler.edu; 2 Department of Mechanical Engineering, New Jersey Institute of Technology, 323 Dr Martin Luther King Jr Blvd, Newark, NJ 07102, USA; leila.donyaparastlivari@njit.edu

**Keywords:** triboelectric, piezoelectric, vibro-impact, hybrid energy harvesting

## Abstract

This study presents a Hybrid Piezoelectric–Triboelectric Energy Harvester (HPTEH) composed of two coupled cantilever beams, designed to enhance energy generation and broaden bandwidth by combining piezoelectric and triboelectric mechanisms. A theoretical 2-DOF lumped model was developed and validated with experimental results, demonstrating good agreement. Experimental findings reveal that Beam I exhibits a softening effect, with resonance frequencies shifting to lower values and increased displacement amplitudes under higher excitation levels due to material nonlinearities and strain-induced voltage generation. Beam II, in contrast, displays a hardening effect, with resonance frequencies increasing as triboelectric interactions enhance stiffness at higher excitation levels. Coupling dynamics reveal asymmetry, with Beam I significantly influencing Beam II in the higher frequency range, while Beam II’s impact on Beam I remains minimal. Phase portraits highlight the dynamic coupling and energy transfer between the beams, particularly near their natural frequencies of 18.6 Hz and 40.6 Hz, demonstrating complex interactions and energy exchange across a broad frequency range. The synergistic interplay between triboelectric and piezoelectric mechanisms allows the HPTEH to efficiently harvest energy across a wider spectrum, underscoring its potential for advanced energy applications in diverse vibrational environments.

## 1. Introduction

Mechanical vibrations counted as a substantial energy source are transformed into electrical energy in a process known as energy harvesting through different transduction mechanisms like piezoelectric, triboelectric, and electromagnetic induction technologies [1].

Energy harvesting technologies play a crucial role in powering small-scale electronic devices by capturing energy from diverse ambient sources [2]. These technologies contribute significantly to industrial operations [3], emergency response [4], home automation [5], and human and structural health monitoring systems [6,7,8]. Despite their potential, vibration energy harvesting systems face notable limitations that affect their efficiency and practicality. These systems often exhibit low energy output [9], and scalability remains a significant challenge, as increasing power output typically requires system enlargement [10]. Moreover, their performance is highly sensitive to resonance conditions [11], necessitating precise tuning to specific frequencies, which restricts their effectiveness in broadband vibration scenarios [12]. To address these limitations, various studies have explored innovative approaches such as vibro-impact mechanisms [13,14,15] and frequency up-conversion techniques [16,17], which enhance energy generation in low-frequency applications and extend the operational bandwidth. Further advancements have been achieved through the optimization of a two-degree-of-freedom (2-DOF) vibratory impact mechanism. This approach leverages multimodality and piecewise linearity between two closely resonant frequencies to significantly improve bandwidth and efficiency [18,19].

Recent research has focused on the creation of hybrid energy harvesting systems, which integrate various harvesting techniques such as solar, thermal, and vibrational to address the issues of intermittency and low power density [20,21,22]. Such hybrid systems can include multiple transduction mechanisms in the same vibrational system to improve the efficiency and range of energy capture [23,24,25,26]. Piezoelectric and triboelectric mechanisms are among the most commonly utilized transduction methods in vibration energy harvesters. However, each mechanism has its advantages and disadvantages. Piezoelectric and triboelectric mechanisms are complementary because piezoelectricity excels at harvesting energy from high-frequency vibrations through strain-induced polarization, while triboelectricity is effective at low-frequency vibrations via contact electrification and electrostatic induction. Combining them in a hybrid system leverages their strengths, enabling efficient energy harvesting across a broader frequency range and diverse mechanical conditions. Moreover, combining the two mechanisms can harness the complementary strengths of each approach, potentially creating a synergistic effect where their combined performance exceeds the sum of their individual contributions of being more efficient, adaptable, and wide operations spectrum compared to single-mechanism systems [27,28,29,30]. Hybrid systems can effectively transform low-frequency mechanical ambient vibrations such as natural and human activities into usable electrical energy [31,32,33], offering a viable solution for powering small-scale sensors and wearable devices [34,35]. For instance, Zhao et al. [36] developed a hybrid triboelectric–electromagnetic energy harvester designed to efficiently capture energy from low-frequency vibrations. Similarly, Wu et al. [37] introduced a hybrid triboelectric–electromagnetic system tailored for harvesting energy from water waves. Li et al. [38] proposed a triboelectric–electromagnetic wind energy harvester capable of powering a thermometer at a wind speed of 8 m/s. Most existing studies have focused on integrating triboelectric energy harvesting (TEH) with electromagnetic energy harvesting (EEH) or piezoelectric energy harvesting (PEH) with EEH within a single system for vibrational energy applications. However, research on combining triboelectric and piezoelectric transducers remains limited, presenting a valuable area for further exploration.

Based on the analysis of previous studies, there remains a gap in understanding the dynamic behavior of hybrid energy harvesters, particularly in terms of frequency response and voltage analysis. This study aims to address this gap by investigating the dynamic behavior of a hybrid cantilever beam energy harvester that integrates piezoelectric and triboelectric transduction mechanisms. The objective is to enhance energy harvesting efficiency by expanding the operational bandwidth and increasing output amplitude at low frequency vibrations below 100 Hz.

## 2. Working Principles

The generation of alternating current (AC) from a vibrating structure with an attached piezoelectric layer under base excitation relies on the piezoelectric effect. When the base of structure is subjected to vibrations, it induces mechanical oscillations in the structure. These oscillations create strain within the piezoelectric layer, causing its internal crystal structure to deform. This deformation generates an electric charge due to the piezoelectric effect, producing an AC as the structure vibrates back and forth. The frequency and amplitude of the resulting AC output correspond to the vibration characteristics of the structure and the mechanical coupling with the base excitation.

**Figure 1:**
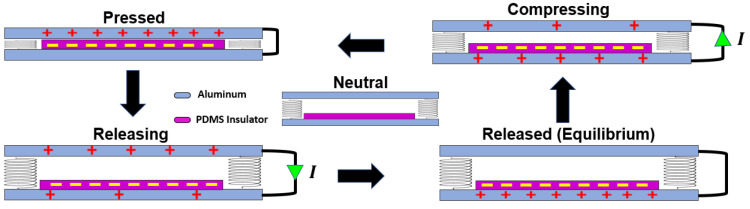
Operating mechanism for the triboelectric energy harvester, where PDMS refers to polydimethylsiloxane.

The triboelectric generator operates through a dual-layer configuration, where each layer possesses distinct tendencies for acquiring or releasing electrons. This system harnesses the principles of contact electrification and electrostatic induction to generate charges. The mechanism involves a cyclic interaction between the layers, which leads to the production of electric charges upon each contact, as shown in Figure 1. The process begins when the two layers make contact, causing electrons to transfer due to the different triboelectric effects of the materials. The polydimethylsiloxane (PDMS) layer, an effective insulator, takes on a negative charge, while the opposing aluminum layer becomes positively charged. This phase of contact electrification is followed by a separation, driven by the system’s inherent mechanical forces, which results in an electron flow between the aluminum electrodes to balance the potential difference, effectively creating an alternating current. This sequence of charging and discharging is maintained by the mechanical dynamics of the system, where the release and re-contact of the layers perpetuate the generation of electrical energy.

## 3. Device Configuration

**Figure 2:**
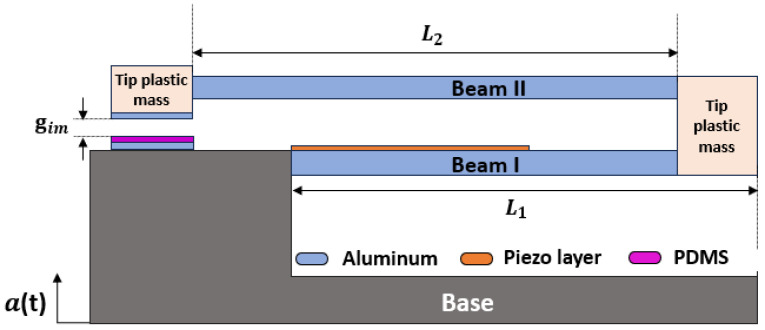
Schematic for the Hybrid Piezoelectric–Triboelectric Energy Harvester (HPTEH) 2-DOF cantilever structure, where PDMS refers to polydimethylsiloxane.

The proposed system is designed as a two-degrees-of-freedom (2-DOF) cantilever beam structure, as shown in Figure 2. This system integrates two distinct energy conversion mechanisms, piezoelectric and triboelectric, forming a Hybrid Piezoelectric–Triboelectric Energy Harvester (HPTEH). The hybrid design aims to expand the operational bandwidth through vibro-impact induced by the triboelectric mechanism and enhance amplitude response via the piezoelectric mechanism.

As depicted in Figure 2, the HPTEH consists of a primary composite cantilever beam constructed from aluminum and integrated with a piezoelectric layer. A tip mass is added at the free end of the primary beam, serving not only as a mass load but also as a base for a secondary cantilever beam made of aluminum, which includes its own tip mass. For simplicity, the primary and secondary beams will be called as Beam I and Beam II, respectively. Beam I, equipped with a piezoelectric transduction mechanism, is mechanically coupled to Beam II, which utilizes a triboelectric transduction mechanism at its free end, through a shared tip mass on Beam I. This geometric connection facilitates interaction and energy transfer between the two beams. The triboelectric harvester features an upper aluminum electrode affixed to the underside of the Beam II tip mass and a lower aluminum electrode mounted on the base. The lower aluminum electrode includes a bonded polydimethylsiloxane (PDMS) insulator, separated from the upper electrode by an air gap, facilitating efficient triboelectric energy generation.

The geometric connection between the two beams enables mechanical coupling, allowing energy to transfer between them through their shared dynamic interactions. When one beam vibrates due to its respective excitation frequency, it induces forces and motions in the other beam via the coupling mechanism. This interaction allows the energy harvested by one mechanism, such as the triboelectric effect, to influence the response of the other, like the piezoelectric effect, and vice versa. This energy exchange enhances the overall system performance by enabling complementary harvesting across different frequency ranges. Upon base excitation, the entire assembly enters a state of oscillation. Beam I experiences bending stresses that activate its piezoelectric layer, converting mechanical strain into an electrical charge through the piezoelectric effect. Simultaneously, the relative motion induced between Beam I and Beam II triggers contact-separation cycles in the triboelectric components of Beam II, generating electrical charges via the triboelectric effect. The dynamic coupling between the beams ensures that the oscillatory behavior of one beam influences the other, creating a synergistic system where energy is harvested efficiently from low-frequency vibrations. This dual transduction approach not only expands the operational bandwidth but also enhances energy harvesting efficiency by leveraging both piezoelectric and triboelectric mechanisms.

## 4. Theoretical Lumped Parameter Model

**Figure 3:**
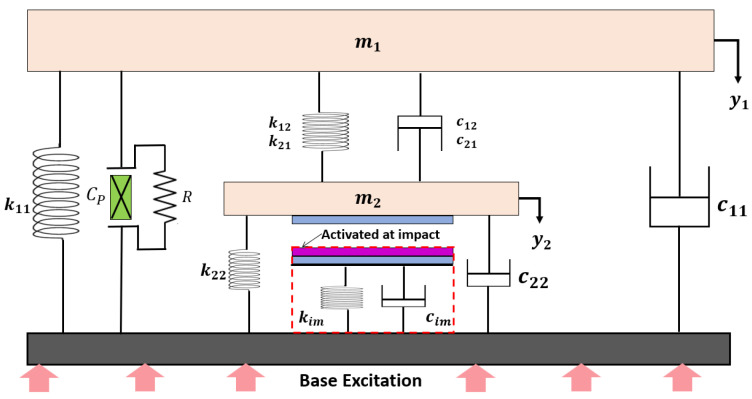
Graphical representation of the 2-DOF spring–mass–damper system with integration of piezoelectric and triboelectric transduction mechanisms.

A lumped parameter model with two degrees of freedom (2-DOF) is utilized to simulate the dynamic responses and electrical output of the harvester. The model is graphically illustrated in Figure 3, depicting a spring–mass–damper system. Under base excitation, the system exhibits two distinct motion patterns: a no-impact mode and an impact mode based on the strength of the excitation level. In the no-impact mode, the excitations are at low level and the tip deflection of the secondary beam remains insufficient to make contact with the PDMS layer. Conversely, the impact mode is activated when the tip displacement exceeds the gap because of higher excitation level, causing periodic contact and separation between the Aluminum electrode and the PDMS layer. This periodic interaction significantly influences both the dynamic behavior of the system and its electrical performance.

According to Figure 3, the governing equations of motion for the 2-DOF system are given by the following general equation:

$$\left[\begin{array}{c} M \end{array}\right]{\ddot{y}}_{i}\left(t\right)+\left[\begin{array}{c} C \end{array}\right]{\dot{y}}_{i}\left(t\right)+\left[\begin{array}{c} K \end{array}\right]y_{i}\left(t\right)=\left[\begin{array}{c} M \end{array}\right]a\left(t\right),\;\;\;i=1,2$$

where *M*, *C*, and *K* are the mass, damping, and stiffness matrices, respectively, and they will be defined later; $y_{i}\left(t\right),i=1,2$ are the relative to the base tip deflections of Beam I and the Beam II, respectively; the dots represent the time derivatives of the deflections. The base excitation described by the function $a\left(t\right)=F_{0}\sin\left(\Omega t\right)$, where $F_{0}$ represents the amplitude of the excitation and Ω the corresponding excitation frequency of the base. The mass matrix is based on the equivalent lumped-parameter model of cantilever beams [39], and can be expressed as:

(1) $$\left[M\right]=\left[\begin{array}{cc} M_{eq1}+M_{eq2} & 0\\ 0 & M_{eq2} \end{array}\right]=\left[\begin{array}{cc} M_{1}+\frac{33m_{1}L_{1}}{140}+M_{eq2} & 0\\ 0 & M_{2}+\frac{33m_{2}L_{2}}{140} \end{array}\right]$$

where $M_{eq1}$ and $M_{eq2}$ are the equivalent masses of Beam I and Beam II, respectively; $M_{1}$ and $M_{2}$ are the tip masses of Beam I and Beam II, respectively; $m_{1}$ and $m_{2}$ are the masses of the Beam I and Beam II, respectively; $L_{1}$ and $L_{2}$ are the lengths of the Beam I and Beam II, respectively. The stiffness matrix is derived based on the stiffness influence coefficients method [40] and written as:

(2) $$\left[K\right]=\left[\begin{array}{cc} k_{11} & k_{12}\\ k_{21} & k_{22} \end{array}\right]=k_{o}\left[\begin{array}{cc} 2E_{1}I_{1}L_{2}^{3}+2E_{2}I_{2}L_{1}\left(3L_{2}^{2}-3L_{1}L_{2}+L_{1}^{2}\right) & E_{2}I_{2}L_{1}^{2}\left(3L_{2}-2L_{1}\right)\\ E_{2}I_{2}L_{1}^{2}\left(3L_{2}-2L_{1}\right) & 2E_{2}I_{2}L_{1}^{3} \end{array}\right]$$

where the scalar $k_{o}$ is defined as:

(3) $$k_{o}=\frac{6E_{1}I_{1}}{4E_{1}I_{1}L_{1}^{3}L_{2}^{3}+3E_{2}I_{2}L_{1}^{4}L_{2}^{2}}$$

Here, $k_{11}$ and $k_{22}$ are the stiffness coefficients of Beam I and Beam II, while $k_{12}$ and $k_{21}$ represent the coupling stiffness between Beam I and Beam II. Similarly $E_{1}I_{1}$ and $E_{2}I_{2}$ denote the bending stiffness of Beam I and Beam II, respectively. The damping matrix is defined as:

$$\left[C\right]=\left[\begin{array}{cc} c_{11} & c_{12}\\ c_{21} & c_{22} \end{array}\right]$$

where, $c_{11}$ and $c_{22}$ are the damping coefficients for Beam I and Beam II, respectively, while $c_{12}$ and $c_{21}$ represent the coupling damping coefficients between the two beams. These coefficients characterize the energy dissipation in the system due to both individual beam dynamics and the interaction between Beam I and Beam II, the equations of motion become:

(4) $$M_{eq1}{\ddot{y}}_{1}+c_{11}{\dot{y}}_{1}+c_{12}{\dot{y}}_{2}+k_{11}y_{1}+k_{12}y_{2}+\theta v=-M_{eq1}a\left(t\right)$$

(5) $$\frac{v}{R}+C_{p}\dot{v}-\theta {\dot{y}}_{1}=0$$

(6) $$\left\{\begin{array}{c} M_{eq2}{\ddot{y}}_{2}+c_{21}{\dot{y}}_{1}+c_{22}{\dot{y}}_{2}+k_{21}y_{1}+k_{22}y_{2}+F_{e}=-M_{eq2}a\left(t\right),\;y_{2}\leq g_{im}\\ M_{eq2}{\ddot{y}}_{2}+c_{21}{\dot{y}}_{1}+c_{22}{\dot{y}}_{2}+k_{21}y_{1}+k_{22}y_{2}+F_{im}=-M_{eq2}a\left(t\right),\;y_{2}>g_{im} \end{array}\right.$$

where *v* is the voltage across the system, *R* is the resistance, $C_{p}$ is the piezoelectric capacity, θ is the piezoelectric coupling coefficient, $g_{im}$ is the air gap between the PDMS and the upper aluminum electrode, and $F_{e}$ is the electrostatic force and can be expressed as [41]:

(7) $$F_{e}=\frac{q{\left(t\right)}^{2}}{2\epsilon_{r}\epsilon_{0}S}$$

where q(t) is the electric charge, $\epsilon_{0}$ and $\epsilon_{r}$ are the permittivity of free space and the PDMS, respectively, and *S* is the contact area. During the impact scenario, the system introduces the impact force into the model. A piecewise function is employed to differentiate between the two scenarios. The impact force is given by:

(8) $$F_{im}=k_{im}\left(y_{2}-g_{im}\right)+c_{im}{\dot{y}}_{2}$$

where $k_{im}$ and $c_{im}$ are the impact stiffness and damping. Finally, the electromechanical coupling equation can be expressed as [41]:

(9) $$\dot{q}\left(t\right)=-\frac{q(t)}{\epsilon_{0}RS}\left(\frac{T}{\epsilon_{r}}+d-y_{2}\right)+\frac{\sigma }{\epsilon_{0}R}\left(d-y_{2}\right)$$

where *T* is the thickness of the PDMS layer, σ is the surface charge density, and *d* is the initial gap distance between two aluminum electrodes when the structure is steady and given by: $d=g_{im}+T$.

### Simulated Linear Analysis

To investigate the dynamic behavior of the Hybrid Piezoelectric–Triboelectric Energy Harvester (HPTEH), this section focuses on analyzing the linear dynamic behavior of the system using the theoretical model derived previously which provides the foundation for understanding the coupled dynamics of the hybrid system, enabling the evaluation of its performance under various excitation conditions. To ensure clarity and reproducibility, the detailed specifications of the device geometry, material properties, and dimensional parameters utilized in this study are systematically presented in Table 1.

**Figure 4:**
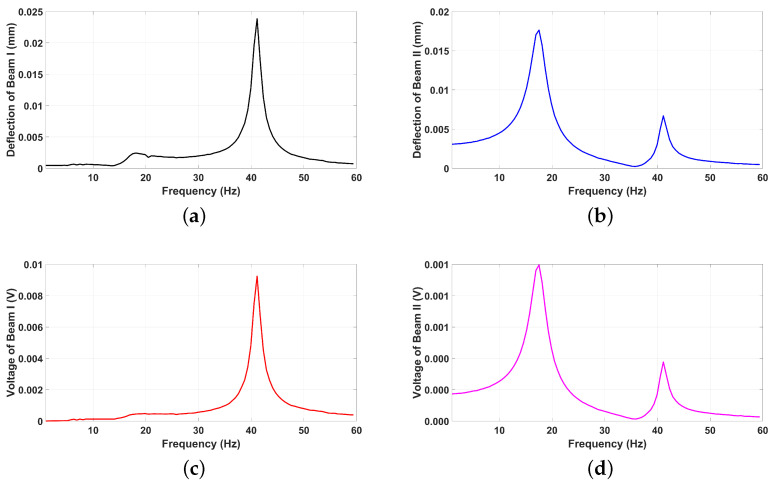
Frequency response and voltage curves for the HPTEH at 0.005 g excitation level: (**a**) Deflection of Beam I, (**b**) Deflection of Beam II, (**c**) Voltage of Beam I, and (**d**) Voltage of Beam II.

These specifications form the basis for the subsequent analysis, offering insights into the interplay between the piezoelectric and triboelectric transduction mechanisms and their contribution to the overall energy harvesting efficiency. Toward this objective, the system was numerically integrated by varying the excitation frequency under a minimal excitation level of 0.005 g. This approach was employed to extract the linear frequency response and frequency–voltage characteristics for both Beam I and Beam II. The result of the frequency response and voltage analysis for Beam I and Beam II exhibit two distinct resonance peaks at 18.6 Hz and 40.6 Hz, corresponding to the natural frequencies of Beam II and Beam I, respectively. These natural frequencies represent points of resonance where the beams experience significant oscillations, even under relatively small excitation levels. At these frequencies, both deflection and voltage responses are notably amplified due to the resonance effect. Also, since Beam I is shorter and inherently stiffer than Beam II, it demonstrates a higher natural frequency of (40.6 Hz). This is also supported by the fact that Beam II is longer and has a big tip mass, which substantially reduces the effective stiffness of Beam II, thereby lowering its natural frequency compared to that of Beam I. The data are presented in Figure 4.

The coupling between Beam I and Beam II is evident in their frequency response curves, where the dynamic interaction significantly influences the resonance behavior of each beam. In the frequency response of Beam I shown in Figure 4a, the effect of Beam II is observed through an amplified peak at Beam II’s natural frequency (18.6 Hz). Similarly, in the frequency response of Beam II in Figure 4b, an amplified peak appears at Beam I’s natural frequency (40.6 Hz). This mutual amplification, particularly pronounced at the low excitation level of 0.005 g, highlights the strong coupling between the two beams. This dynamic interaction is further reflected in the corresponding frequency voltage curves as illustrated in Figure 4c,d, where the voltage outputs for both beams show amplified responses at the natural frequencies of their coupled counterparts. These observations confirm the robust energy transfer and interaction between the piezoelectric and triboelectric mechanisms facilitated by the coupling of the beams.

The linear dynamic analysis of the Hybrid Piezoelectric–Triboelectric Energy Harvester (HPTEH) provides a fundamental understanding of the coupled behavior between the piezoelectric and triboelectric mechanisms. This analysis highlights the strong coupling effects and their role in amplifying energy harvesting efficiency at specific frequencies. By examining the system’s response under varying excitation conditions, valuable insights into resonance interactions and energy transfer between the beams can be obtained.

## 5. Experimental Setup

**Figure 5:**
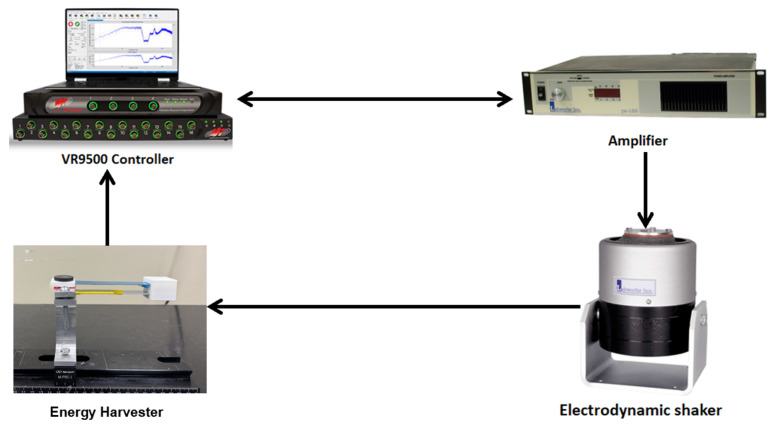
The experimental setup for testing the HPTEH.

To validate the theoretical model and to evaluate the dynamic behavior of the HPTEH, experiments were conducted using the setup depicted in Figure 5. The experimental setup comprises a Vibration Research (VR) controller (VR9500) [42], an amplifier and an electrodynamic shaker [43] that supports the energy harvester structure. The controller regulates the excitation amplitude and frequency of the electrodynamic shaker. A closed-loop system between the controller and the amplifier ensures that the excitation level remains consistent while the frequency is swept across the desired range. The dynamic response of the structure is measured using accelerometers mounted on the tip masses of the beams, and the voltage output from each transduction mechanism is recorded directly by the controller.

## 6. Results and Discussion

### 6.1. Experimental Results

**Figure 6:**
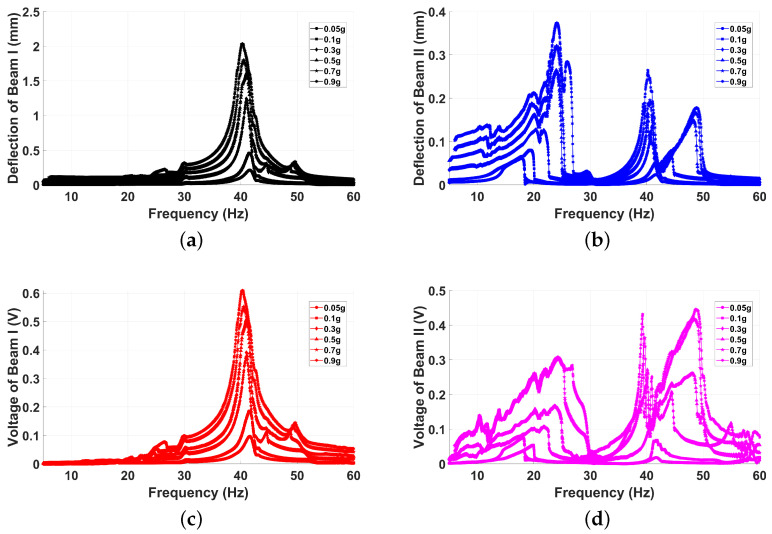
Experimental frequency response and voltage curves for the HPTEH at different excitation level: (**a**) Deflection of Beam I, (**b**) Deflection of Beam II, (**c**) Voltage of Beam I, and (**d**) Voltage of Beam II.

The experimental results presented in this section were obtained using the setup detailed in the previous section to evaluate the performance of the HPTEH structure under varying excitation conditions. The structure was tested by systematically sweeping the excitation frequency around the natural frequencies of Beam I and Beam II while varying the excitation levels from 0.05 g to 0.9 g. The frequency sweep ranged from 5 Hz to 60 Hz, encompassing the expected resonance frequencies of both beams. The resulting frequency response and the corresponding frequency–voltage characteristics for Beam I and Beam II are illustrated in Figure 6.

The energy transfer between the piezoelectric and triboelectric harvesters arises from their coupling through the shared structural dynamics of the hybrid system. The two harvesters exploit distinct physical phenomena: the piezoelectric harvester generates energy from mechanical strain-induced polarization in piezoelectric materials, while the triboelectric harvester relies on contact electrification and electrostatic induction. In the hybrid configuration, these two mechanisms are interconnected through the shared base excitation and dynamic response of the structure. When the system is excited, vibrations induce deformation and relative motion, simultaneously activating the piezoelectric and triboelectric mechanisms. This dynamic interaction causes energy to be distributed across the system, with each harvester responding to specific frequency ranges. The coupling effect amplifies the overall energy harvesting performance by broadening the operational bandwidth and enhancing the total energy output. This synergy between the harvesters is a key advantage of the hybrid design, leveraging the complementary characteristics of the two mechanisms to achieve superior energy harvesting efficiency.

The displacement amplitudes across varying excitation levels ranging from 0.05 g to 0.9 g for Beam I is illustrated in Figure 6a. A distinct trend is observed where the amplitude of displacement increases with higher g-levels, indicating that greater excitation contributes to increased energy transfer and deformation of the piezoelectric material. Furthermore, the resonance frequency, characterized by the peak in displacement amplitude, exhibits a slight shift towards lower frequencies as the g-level increases. This shift suggests the presence of a softening effect, where the effective stiffness of the system decreases under larger excitations, potentially due to material nonlinearities and boundary condition changes. The softening effect in the displacement response becomes more clear at higher g-levels, indicating the complex mechanical interactions within the piezoelectric element. The corresponding voltage output at the same excitation levels is shown in Figure 6c demonstrates a similar pattern, with voltage peaks occurring at the resonance frequencies for each g-level. The voltage output increases with higher excitation levels, reflecting the strain-induced voltage generation characteristic of piezoelectric materials. Also, the resonance frequency again exhibits a softening behavior, as it decreases with increased g-levels. Additionally, at higher g-levels, the voltage output shows signs of saturation, where the increases in the voltage output is not significant compared to the lower excitations, indicating that the piezoelectric material may be getting close to reach its maximum strain capacity, beyond which further increases in excitation do not yield significant voltage gains. This saturation effect highlights the material limitations in high-excitation scenarios.

Regarding the coupling dynamics, the influence of Beam II on Beam I appears to be minimal, as evidenced by the relatively small amplitude response observed near Beam II’s natural frequency (18.6 Hz), as shown in Figure 6a,c. This indicates that the coupling effect from Beam II on Beam I is weak, resulting in limited energy transfer or interaction at this specific frequency. The subdued amplitude suggests that the mechanical and vibrational influence of Beam II is insufficient to significantly alter the dynamic behavior of Beam I around its resonance. This observation underscores the asymmetry in coupling strength between the two beams within the hybrid system.

The displacement of Beam II, as shown in Figure 6b, exhibits a more complex behavior compared to the piezoelectric system for Beam I. Multiple peaks are observed, resulting from the intricate interplay between mechanical vibrations and the impact phenomenon from the triboelectric harvester. These multiple frequencies indicate a more diverse vibrational response in the triboelectric system, driven by dynamic contact and separation processes. Unlike the softening behavior of Beam I, the resonance frequencies in Beam II frequency response increase with higher excitation levels, suggesting a hardening effect. This behavior, characteristic of systems where effective stiffness rises with excitation, may stem from enhanced frictional forces and increased contact pressure at higher g-levels. Similarly, the voltage output of the triboelectric harvester of Beam II, depicted in Figure 6d, mirrors this trend, with multiple peaks corresponding to distinct resonance frequencies.

The increase in resonance frequencies with higher g-levels further confirms the hardening effect observed in the displacement response. This behavior is likely influenced by augmented contact forces and surface interactions, leading to a stiffer system response at elevated excitation levels. Moreover, the variability in voltage output across different g-levels highlights the triboelectric system’s sensitivity to surface contact conditions. Factors such as surface roughness, contact pressure, and environmental conditions significantly affect charge transfer efficiency, contributing to the observed variations in voltage performance.

Furthermore, the results clearly demonstrate a significant increase in bandwidth around Beam II’s natural frequency range (18.6 Hz), primarily due to the impact within the triboelectric layers, which occurs over a broader frequency range. This extended bandwidth greatly enhances the overlap with ambient vibrations, thereby improving the harvester’s efficiency and the total energy harvested. At higher frequencies, near Beam I’s natural frequency (40.6 Hz), the influence of Beam I on Beam II becomes notably significant. This is evident from the relatively large amplitude response observed near Beam I’s resonance frequency (40.6 Hz), as well as the interaction and strong coupling between the two beams in the higher frequency range (35–50 Hz). Despite the triboelectric effect occurring predominantly at lower frequency range (10–30 Hz), the coupling effect from Beam I on Beam II facilitates a wider bandwidth in the higher frequency range. This strong coupling leads to substantial energy transfer and interaction across broader frequency ranges. The high amplitudes observed are attributed to the robust mechanical interplay and vibrational influence between the two beams, which significantly modify their dynamic behavior around their respective resonance frequencies.

The experimental results reveal key insights into the coupling behavior between the piezoelectric and triboelectric harvesters. While the voltage drop between resonance peaks may initially suggest a low coupling between the two harvesters, this observation reflects the inherent differences in their dynamic responses and operational frequency ranges. The piezoelectric and triboelectric mechanisms operate optimally at distinct frequencies due to their individual physical principles, which is a deliberate design feature of the hybrid system. The primary goal of the coupling is not to eliminate these gaps but to achieve a broader operational bandwidth by leveraging the complementary frequency ranges of the two harvesters.

Additionally, the displacement results are influenced by the structural dynamics and the specific excitation levels tested during the experiments. The coupling between the harvesters enables the system to transfer energy effectively across a wider range of frequencies, even if individual harvesters show varying performance at certain points. To emphasize the improvement in energy harvesting performance, we focused the energy across the combined bandwidth rather than focusing solely on peak voltages. The hybrid design intentionally creates a system where the strengths of both harvesters are utilized to achieve enhanced overall performance, as evident in the broadened frequency response and increased operational range. This coupling effect underscores the value of combining piezoelectric and triboelectric mechanisms in a single system.

### 6.2. Validation of the Theoretical Model

**Figure 7:**
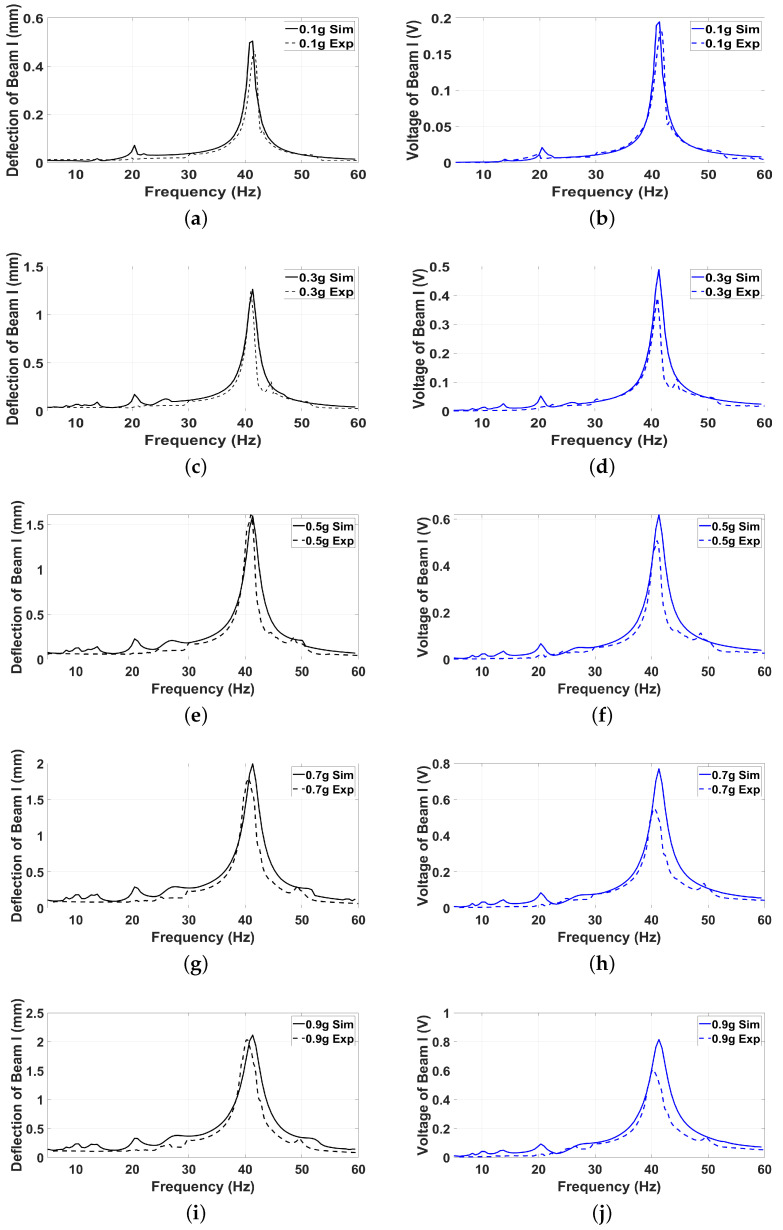
The experimental and simulated frequency-response (**a**,**c**,**e**,**g**,**i**) and frequency-voltage (**b**,**d**,**f**,**h**,**j**) curves of Beam I at different excitation levels.

**Figure 8:**
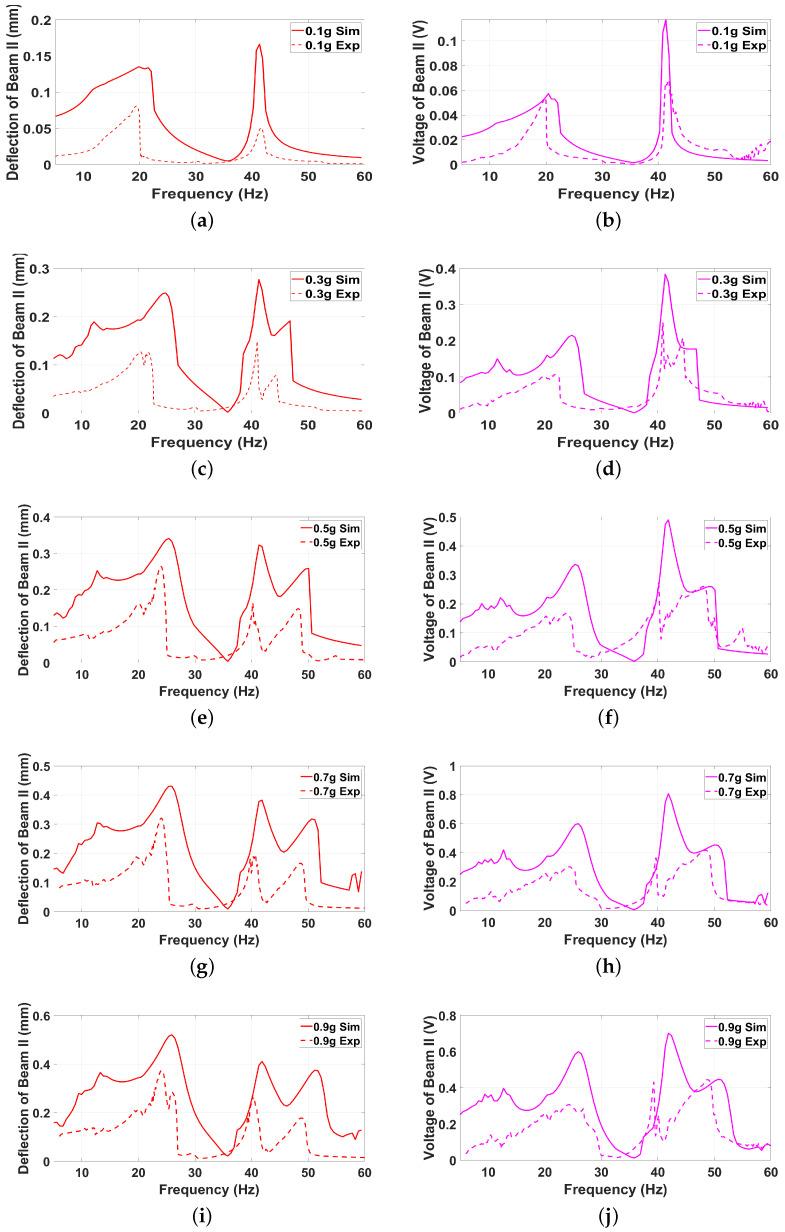
The experimental and simulated frequency-response (**a**,**c**,**e**,**g**,**i**) and frequency-voltage (**b**,**d**,**f**,**h**,**j**) curves of Beam II at different excitation levels.

To validate the theoretical model developed in earlier sections, this section presents a comparative analysis between the simulated results and the experimental findings discussed previously. The validation process aims to assess the accuracy and reliability of the theoretical predictions by examining key performance metrics such as displacement amplitudes, resonance frequencies, and voltage outputs for both Beam I and Beam II. By aligning the simulation data with the experimentally observed dynamic behavior, the robustness of the theoretical framework can be evaluated. This comparison not only highlights the strengths of the model but also identifies any discrepancies, offering valuable insights for further refinement and optimization of the Hybrid Piezoelectric–Triboelectric Energy Harvester (HPTEH) design. The comparison between the experimental and simulated results for selected excitation levels of 0.1 g, 0.3 g, 0.5 g, 0.7 g, and 0.9 g for Beam I and Beam II shown in Figure 7 and Figure 8, respectively, demonstrates a strong agreement, validating the robustness of the theoretical model in capturing the key dynamic behaviors of the Hybrid Piezoelectric–Triboelectric Energy Harvester (HPTEH).

Both the frequency response and voltage output curves align closely across these g-levels, accurately reflecting the resonance frequencies and the general trends of amplitude and voltage variations. The model successfully captures critical phenomena such as the softening effect in Beam I and the hardening effect in Beam II, as well as the coupling interactions between the two beams. These findings confirm that the derived theoretical framework effectively represents the hybrid system’s dynamics under a wide range of excitation conditions.

However, some discrepancies between the experimental and simulated results are observed, particularly in the amplitude magnitudes and the exact locations of certain resonance peaks. These deviations can be attributed to several factors. First, the theoretical model employs a simplified lumped-parameter approach, which inherently assumes idealized conditions and may not fully capture the distributed mass, stiffness variations, and boundary effects of the actual system. Second, the triboelectric interactions in the experimental setup are highly sensitive to surface properties, such as roughness and contact pressure, which may vary slightly during the experiment and introduce inconsistencies not accounted for in the model. Third, minor experimental errors, including measurement inaccuracies from sensors, noise in the recorded signals, or slight misalignments in the setup, could also contribute to the observed differences. Finally, environmental factors, such as temperature fluctuations or air damping, might influence the experimental results but are not explicitly incorporated into the model.

Despite these minor discrepancies, the overall agreement between the experimental and simulated results highlights the efficacy of the theoretical model as a reliable tool for predicting the dynamic performance of the HPTEH. These insights can guide further refinements to the model, incorporating more detailed physical representations or mitigating experimental uncertainties to achieve even greater precision.

The results presented in Table 2 demonstrate the power generated by the piezoelectric and triboelectric harvesters at varying excitation levels (g-levels). As the g-level increases from 0.1 g to 0.9 g, the power output for both mechanisms shows a noticeable increase, indicating a positive correlation between excitation amplitude and energy harvesting efficiency. The piezoelectric harvester exhibits a consistently higher power output compared to the triboelectric harvester across all g-levels, with the highest power recorded at 0.9 g being 0.664 μW. On the other hand, the triboelectric harvester generates a peak power of 0.653 μW at 0.7 g, followed by a slight decrease at 0.9 g, which may suggest saturation effects or nonlinear behavior in the triboelectric system. These results highlight the complementary nature of the two mechanisms, where the piezoelectric harvester excels at higher g-levels, while the triboelectric harvester contributes significantly under varying conditions, enhancing the overall energy harvesting performance of the hybrid system.

The operational range of the dynamic behavior for the HPTEH spans from 5 Hz to 60 Hz, demonstrating its potential for wideband energy harvesting. To further enhance the bandwidth, the excitation level can be increased, enabling the combination of the triboelectric harvester’s bandwidth (10–26 Hz) with the piezoelectric harvester’s bandwidth (40–50 Hz) to achieve an integrated wideband response from 10 Hz to 50 Hz. Alternatively, modifications such as adjusting the length of the piezoelectric beam or slightly increasing the tip mass can effectively lower its natural frequency, facilitating a more seamless overlap between the two harvesters’ operational ranges and achieving a broader bandwidth for improved performance.

### 6.3. Non-Linear Analysis

Building on the validation of our theoretical model in the previous section, we now extend our investigation to explore the dynamic behavior of both beams in the Hybrid Piezoelectric–Triboelectric Energy Harvester (HPTEH) using phase portrait analysis. Phase portraits provide a powerful tool for visualizing the trajectories of the system in state space, offering insights into the nature of the oscillations and their stability. To achieve this, we focus on an excitation level of 0.5 g and examine the system’s response at various excitation frequencies. By plotting the phase portraits of both beams, we aim to uncover the intricate interplay between the piezoelectric and triboelectric mechanisms and their dynamic characteristics under resonance and off-resonance conditions. This analysis will deepen our understanding of the system’s behavior and its potential for efficient energy harvesting.

For the 0.5 g excitation results of Beam I, depicted in Figure 7e, the dynamic interaction between Beam I and Beam II is evident across a wide frequency range, starting from approximately 10 Hz and extending up to 50 Hz. This interaction suggests significant coupling effects and energy exchange between the two beams within this frequency band. To further analyze this behavior, specific frequencies within this range were selected to investigate the system’s dynamics in detail using phase portraits for both beams. These phase portraits provide a visualization of the displacement-velocity relationship, highlighting the intricate interactions and oscillatory patterns of the system. According to the results in Figure 7e, Beam I and Beam II exhibit natural frequencies of 18.6 Hz and 40.6 Hz, respectively. Their coupling introduces significant interaction effects that shape their motion, particularly near resonance frequencies. The corresponding phase portraits of a 2-DOF coupled system, consisting of Beam I and Beam II, at 0.5 g excitation level and various excitation frequencies are summarized in Table 3.

The phase portraits of Beam I and Beam II under 10 Hz excitation reveal distinct dynamics influenced by their respective energy harvesting mechanisms and coupling effects. Beam I’s phase portrait exhibits irregular, non-elliptical loops, indicating non-resonant behavior. The displacement and velocity amplitudes remain small, as the excitation frequency is far below Beam I’s natural frequency of 18.6 Hz. The irregularity in the loops suggests nonlinear effects, likely caused by the dynamic coupling with Beam II. This interaction slightly modulates Beam I’s motion, resulting in weak energy absorption by the piezoelectric harvester, which is more effective near resonance. In contrast, Beam II’s phase portrait displays a more complex and distorted trajectory, characterized by elongated loops and noticeable asymmetry. This behavior reflects the triboelectric harvester’s reliance on impact-based mechanisms, which involve dynamic contact and separation processes. The larger displacement and velocity amplitudes of Beam II, compared to Beam I, suggest higher sensitivity to the excitation frequency. The coupling with Beam I contributes to the irregularity of the loops, as energy transfer between the beams influences Beam II’s motion. Additionally, the intermittent contact inherent in the triboelectric mechanism introduces nonlinearities, further distorting the phase portrait and emphasizing the complex dynamics of the system.

As the excitation frequency increases to 15 Hz, the phase portrait of Beam I reveals slightly larger loops, signaling an increase in energy transfer as the system moves closer to Beam I’s natural frequency (18.6 Hz). This behavior suggests the onset of dynamic coupling between the two beams. Beam II’s phase portrait also exhibits moderate distortion, with slightly larger amplitudes compared to 10 Hz. This implies that the interaction between the beams begins to influence Beam II’s motion more noticeably.

At an excitation frequency of 20 Hz, the phase portraits highlight distinct behaviors for Beam I and Beam II based on their natural frequencies of 40.6 Hz and 18.6 Hz, respectively. Beam II, with a natural frequency close to the excitation frequency, exhibits near-resonance behavior, as seen in the distorted elliptical phase portrait. The significant displacement and velocity amplitudes, combined with the elongated and asymmetric shape, indicate the dominance of Beam II in energy absorption at this frequency. The nonlinearities in its response, arising from the triboelectric harvester’s impact-based mechanism, are evident through the irregularities in the phase portrait, reflecting dynamic contact-separation interactions. In contrast, Beam I’s phase portrait shows smaller, regular elliptical loops, characteristic of sub-resonant behavior. With the excitation frequency far below its natural frequency, Beam I’s response remains linear, with limited displacement and velocity amplitudes, indicating reduced energy absorption at this frequency.

At 30 Hz, the phase portraits of both beams exhibit regular elliptical shapes, albeit with reduced amplitudes compared to 20 Hz. Beam I’s response is off-resonance but still influenced by its interaction with Beam II. Similarly, Beam II shows moderate amplitudes, suggesting that dynamic coupling between the beams persists, albeit less pronounced than at their natural frequencies.

At 43 Hz, Beam II reaches very close to its natural frequency, as evident from its large, nearly perfect elliptical phase portrait. This resonance condition results in maximum displacement and velocity for Beam II, as it absorbs energy efficiently. In contrast, Beam I’s phase portrait displays moderate amplitudes with an elliptical shape, indicating that it is off-resonance but significantly influenced by the strong coupling with Beam II.

At 50 Hz, the phase portraits for both beams show smaller, regular elliptical loops. Beam I’s reduced amplitudes reflect weak coupling effects as the excitation frequency moves further from its natural frequency. Similarly, Beam II’s response exhibits reduced amplitudes, indicative of off-resonance behavior. The coupling effect diminishes as the excitation frequency deviates further from the resonance frequencies of both beams.

In summary, the phase portraits demonstrate the dynamic coupling and energy transfer between Beam I and Beam II. Strong coupling effects are evident near the natural frequencies of each beam, particularly at 18.6 Hz for Beam I and 40.6 Hz for Beam II. These interactions result in complex dynamic behavior, with noticeable energy exchange even at intermediate frequencies. The irregular shapes observed at lower frequencies suggest potential nonlinearities in the system, influenced by material properties, damping, or boundary conditions. This analysis highlights the importance of coupling in shaping the overall dynamic response and energy distribution within the 2-DOF system.

## 7. Conclusions

This study describes the bandwidth broadening and enhancement of voltage amplitude of vibrational energy harvesters when combining piezoelectric and triboelectric mechanisms in a HPTEH of two coupled cantilever beams. The experimental results provide a comprehensive understanding of the dynamic behavior of the HPTEH system under varying excitation levels. Beam I exhibits a softening effect, with resonance frequencies shifting to lower values and displacement amplitudes increasing with higher excitation levels, reflecting the material’s nonlinearities and strain-induced voltage generation. Conversely, Beam II demonstrates a hardening effect, with resonance frequencies increasing due to enhanced stiffness from triboelectric interactions at higher excitation levels. The coupling dynamics reveal asymmetry, with Beam I significantly influencing Beam II in the higher frequency range (35–50 Hz), while Beam II’s effect on Beam I remains minimal. In addition, the phase portraits underscore the significant dynamic coupling and energy transfer between Beam I and Beam II, particularly near their natural frequencies of 18.6 Hz and 40.6 Hz. The observed interactions reveal complex dynamic behaviors and energy exchange across a range of frequencies, emphasizing the role of coupling in shaping the system’s overall response and energy distribution. The theoretical 2-DOF lumped model was validated and show a good agreement with the experimental results. The increased bandwidth and interaction facilitated by the triboelectric layers at lower frequencies and the piezoelectric coupling at higher frequencies highlight the hybrid system’s ability to efficiently harvest energy across a broader frequency range, maximizing performance under diverse ambient vibrations. These findings underscore the potential of hybrid energy harvesters for advanced energy applications. 

## Figures and Tables

**Table 1:** Geometric and physical parameters of the model.

Name	Symbol	Beam I	Beam II	Tribo.	Piezo.
Length (cm)	$L_{i}$	8.7	12	2	7
Width (cm)	$w_{i}$	2	2	2	2
Thickness (mm)	$h_{i}$	1.5	1	0.5	0.5
Young’s modulus (GPa)	$E_{i}$	69	69	-	-
Density (kg/m^3^)	$\rho_{i}$	2700	2700	-	-
Tip mass (g)	$M_{i}$	11	11	-	-
Damping (Ns/m)	$c_{ii}$	$c_{11}=0.28$	$c_{22}=0.22$	-	-
Average Coupling Damping	$c_{ij}$	$c_{12}=0.12$	$c_{21}=0.12$	-	-
Impact Damping (Ns/m)	$c_{im}$	-	3000	-	-
Impact Stiffness (N/m)	$k_{im}$	-	100	-	-
Space permittivity	$\varepsilon_{0}$	-	-	$8.854\times 10^{-12}$	-
Dielectric constant	$\varepsilon_{r}$	-	-	0.0001	-
Air gap (mm)	$g_{im}$	-	-	0.4	-
Average Surface Charge Density (C/m^2^)	σ	-	-	$0.25\times 10^{-8}$	-
Piezoelectric constant (mm/V)	θ	-	-	-	0.15
Piezoelectric capacity ( nF )	$C_{p}$	-	-	-	72
Resistance (MΩ)	$R_{i}$	-	-	1	1

**Table 2:** Power generated by piezoelectric and triboelectric harvesters at different g-levels.

g-Level	Piezoelectric Power (μW)	Triboelectric Power (μW)
0.1 g	0.038	0.014
0.3 g	0.239	0.147
0.5 g	0.382	0.240
0.7 g	0.593	0.653
0.9 g	0.664	0.492

**Table 3:** Phase plots of Beam I and Beam II at different excitation frequencies.

Description	Beam I	Beam II
10 Hz	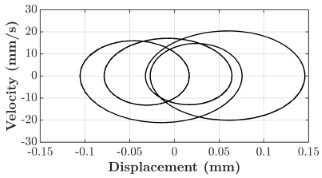	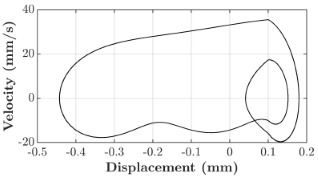
15 Hz	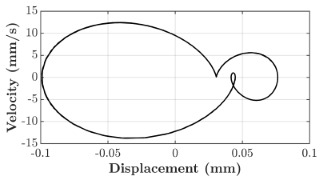	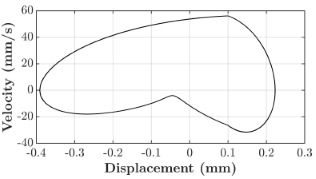
20 Hz	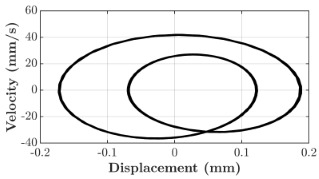	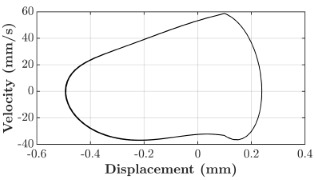
30 Hz	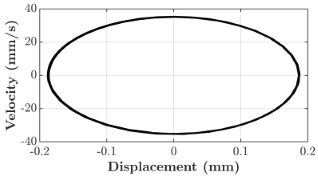	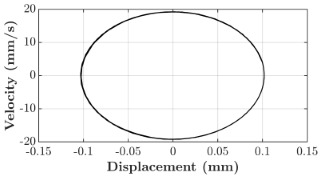
43 Hz	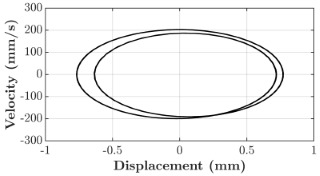	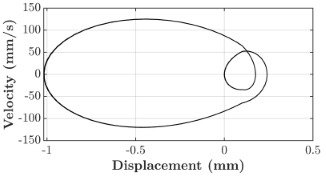
50 Hz	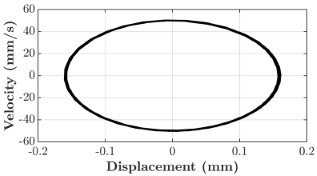	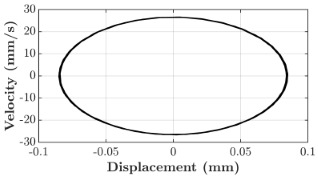

## Data Availability

Data are available on request from the authors.
